# Deep learning of mammogram images to reduce unnecessary breast biopsies: a preliminary study

**DOI:** 10.1186/s13058-024-01830-9

**Published:** 2024-05-24

**Authors:** Chang Liu, Min Sun, Dooman Arefan, Margarita Zuley, Jules Sumkin, Shandong Wu

**Affiliations:** 1 Department of Bioengineering, University of Pittsburgh, Pittsburgh, PA 15213 USA; 2 Hillman Cancer Center, University of Pittsburgh Medical Center, Pittsburgh, PA 15215 USA; 3 Department of Radiology, University of Pittsburgh School of Medicine, Pittsburgh, PA 15213 USA; 4 Magee-Womens Hospital, University of Pittsburgh Medical Center, Pittsburgh, PA 15213 USA; 5 Department of Biomedical Informatics, University of Pittsburgh, Pittsburgh, PA 15213 USA; 6 Intelligent Systems Program, University of Pittsburgh, Pittsburgh, PA 15213 USA

## Abstract

**Background:**

Patients with a Breast Imaging Reporting and Data System (BI-RADS) 4 mammogram are currently recommended for biopsy. However, 70–80% of the biopsies are negative/benign. In this study, we developed a deep learning classification algorithm on mammogram images to classify BI-RADS 4 suspicious lesions aiming to reduce unnecessary breast biopsies.

**Materials and methods:**

This retrospective study included 847 patients with a BI-RADS 4 breast lesion that underwent biopsy at a single institution and included 200 invasive breast cancers, 200 ductal carcinoma in-situ (DCIS), 198 pure atypias, 194 benign, and 55 atypias upstaged to malignancy after excisional biopsy. We employed convolutional neural networks to perform 4 binary classification tasks: (I) benign vs. all atypia + invasive + DCIS, aiming to identify the benign cases for whom biopsy may be avoided; (II) benign + pure atypia vs. atypia-upstaged + invasive + DCIS, aiming to reduce excision of atypia that is not upgraded to cancer at surgery; (III) benign vs. each of the other 3 classes individually (atypia, DCIS, invasive), aiming for a precise diagnosis; and (IV) pure atypia vs. atypia-upstaged, aiming to reduce unnecessary excisional biopsies on atypia patients.

**Results:**

A 95% sensitivity for the “higher stage disease” class was ensured for all tasks. The specificity value was 33% in Task I, and 25% in Task II, respectively. In Task III, the respective specificity value was 30% (vs. atypia), 30% (vs. DCIS), and 46% (vs. invasive tumor). In Task IV, the specificity was 35%. The AUC values for the 4 tasks were 0.72, 0.67, 0.70/0.73/0.72, and 0.67, respectively.

**Conclusion:**

Deep learning of digital mammograms containing BI-RADS 4 findings can identify lesions that may not need breast biopsy, leading to potential reduction of unnecessary procedures and the attendant costs and stress.

## Introduction

Breast cancer is the most common malignancy of females in the United States [1]. Mammography is widely used for screening to detect early breast cancer and the benefits have been shown in multiple clinical trials [1, 2]. One of the concerns of mammography screening is the high rate of detected findings recommended for biopsy that are found to be benign. The creation of the Breast Imaging Reporting and Data System (BI-RADS) aimed to enhance the description and appropriate classification of mammographic findings and to enable the monitoring of outcomes to elevate the standard of patient care [3]. Most (70–80%) BI-RADS 4 findings, which indicate a suspicious abnormality for which biopsy is recommended, are found to be benign [3]. It is estimated that over 970,000 breast biopsies are unnecessary in the United States annually [4]. The high false positive rate increases patient anxiety/stress, clinical procedures, and medical costs.

Computer-aided diagnosis has been proposed to improve breast cancer diagnosis through the analysis of mammograms [5]. Recent advancements in deep learning techniques empower the implementation of computer-aided diagnostic models [6]. The ever-increasing computational capacity and the availability of big data offer unprecedented opportunities for deep learning modeling in multiple image classification tasks [7–9]. Unlike the use of hand-crafted imaging features, deep learning models extract image features automatically through convolutional neural networks (CNNs) [6]. CNN has been shown to be effective for various breast imaging applications [7], such as risk assessment [10], breast tumor detection [11], and breast density classification [12]. In this study, the purpose was to build deep learning models using digital mammograms to predict biopsy outcomes for BI-RADS 4 lesions, aiming at reducing unnecessary biopsy rates for patients who do not have breast cancer.

## Methods and materials

### Study cohort and imaging dataset

We conducted an Institutional Review Board (IRB)-approved retrospective study. Informed consent from patients was waived due to the retrospective nature. This study included 847 patients from 2016 to 2018 identified in the general population breast cancer screening at our institution. All patients had a BI-RADS 4 diagnosis and biopsy-proven outcomes, including 194 benign lesions, 198 pure atypia, 200 ductal carcinoma in situ (DCIS), 200 invasive carcinoma, and 55 atypia that were upstaged to malignancy after excisional biopsies. All of the lesions were detected with screening mammography. For each patient, bilateral craniocaudal (CC) and mediolateral oblique (MLO) views of the (diagnostic) mammogram images were collected. Subclassifcation of BI-RADS 4 was being incorporated into our clinical practice during this timeframe. This is why some cases are rated as 4 without subset classification and others have subclass information. All mammographic examinations were acquired by Hologic/Lorad Selenia (Marlborough, MA) full-field digital mammography units.

### Classification tasks for biopsy outcome prediction

**Fig. 1:**
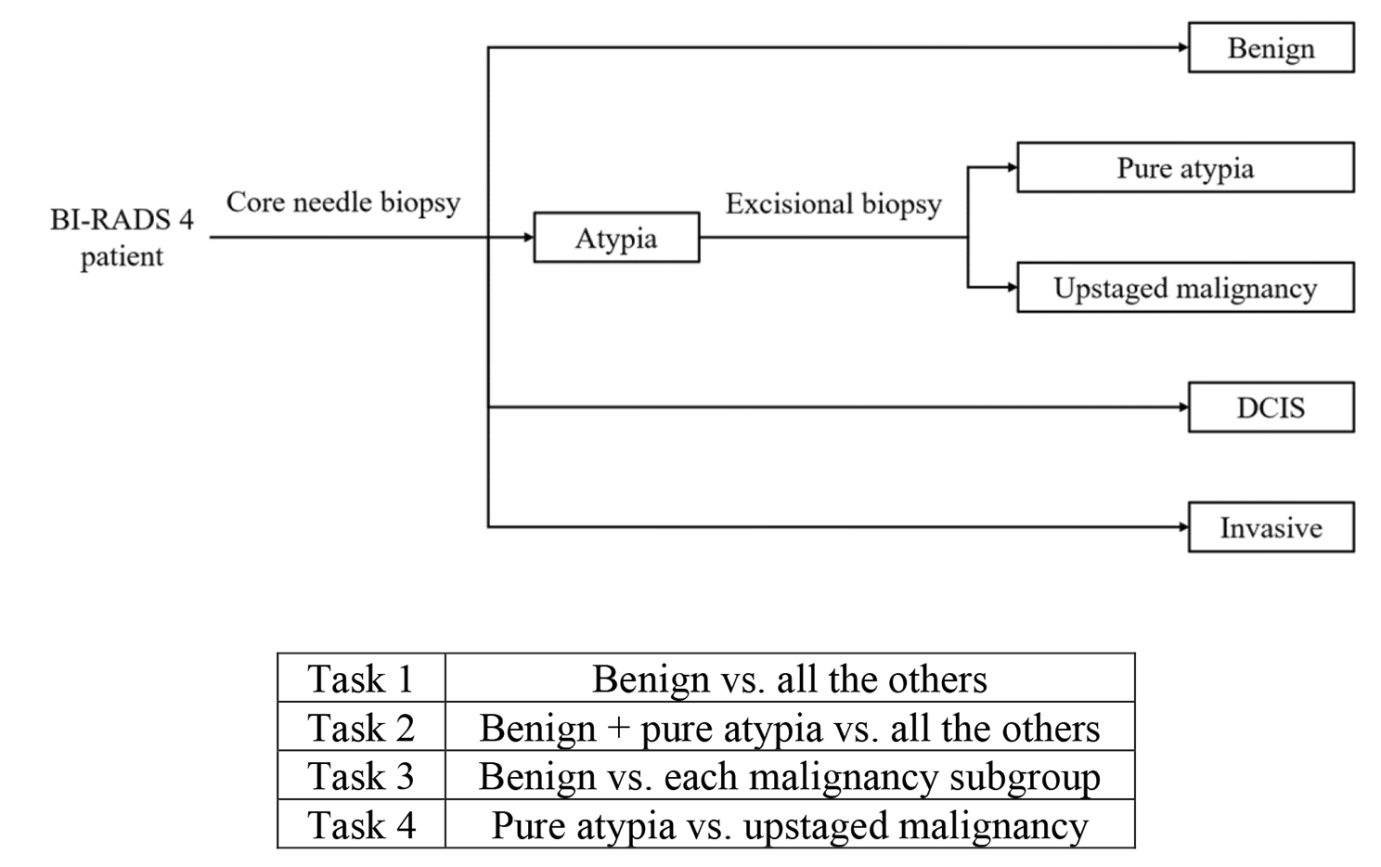
Clinical workflow of the BI-RADS 4 patients and the 4 classification tasks

For BI-RADS 4 patients, standard-of-practice starts with core needle biopsy for tissue diagnosis. If an atypia is diagnosed on the initial core biopsy, an excisional biopsy will be performed to further determine the presence of DCIS or invasive malignancy. Based on the clinical workflow, we designed the four binary classification tasks below (shown in Fig. 1) to classify/predict the biopsy outcome for the BI-RADS 4 lesions.

#### Task 1

To distinguish patients with benign lesions from all the other lesions (i.e., benign vs. atypia, invasive, and DCIS), aiming to identify the potential benign cases for which core needle biopsy may be avoided. In our dataset, this is to classify 194 benign vs. the combination of 198 pure atypia, 55 upstaged malignancy, 200 invasive, and 200 DCIS. This task is directly relevant towards reducing unnecessary breast biopsies.

#### Task 2

To distinguish patients with benign lesions and pure atypia from others (i.e., benign and pure atypia vs. upstaged malignancy, invasive, and DCIS). This is deemed a robustness analysis of Task 1, and it uses a different clinical threshold to distinguish non-malignant vs. malignant lesions. This is to classify the combination of 194 benign and 198 pure atypia vs. the combination of 55 upstaged malignancy, 200 invasive, and 200 DCIS.

#### Task 3

To distinguish patients with benign lesions with each of the malignant outcomes (i.e. benign vs. atypia, benign vs. invasive, and benign vs. DCIS, respectively), aiming for precision diagnosis. This is to classify 194 benign vs. the combination of 198 pure atypia and 55 upstaged malignancy; 194 benign vs. 200 invasive; and 194 benign vs. 200 DCIS.

#### Task 4

To distinguish between cases of pure atypia vs. cases of atypia in patients exhibiting DCIS or invasive malignancy, we rely on excisional biopsy results. In this task, we aim to identify pure atypia patients that would potentially require no additional excisional biopsy. This is to classify 198 pure atypia vs. 55 upstaged malignancy patients.

### Classification with deep learning

We used CNN to build the classification models using mammogram images as input. All mammogram images went through a pre-processing step. First, we ran the LIBRA software package version 1.0.4 (Philadelphia, PA, 2016) [13] to extract the whole breast region (excluding non-breast regions in the images). Then the images were normalized to a fixed intensity range of 0 to 1 and subsequently resampled to the same size of 256 × 256 using the bicubic interpolation algorithm.

The structure of the CNN model was a VGG16 network [14], a 16-layer convolutional neural network. The network was pretrained with a very large non-medical dataset (ImageNet) [15] and then fine-tuned with the training set of our mammogram image dataset. Our CNN model was implemented using Python (version 3.6), TensorFlow (version 1.13), and Keras (version 2.1.6). The model was run on a TitanX Pascal Graphics Processing Unit (GPU) with 12 GB RAM. Adam [16] was employed as the optimizer, with a batch size of 32 and a learning rate of 0.0001. Dropout with a probability of 0.5 was applied during the training procedure. Horizontal flipping was used for data augmentation. To combine CC and MLO view’s prediction, we first generated predictions from individual models on each view with the same data split. Then, we organized each model’s predictions as feature columns, trained a logistic regression model on the training dataset and evaluated its performance on the testing set.

### Statistical analysis

We performed a patient-wise data split for CNN model training and testing. For Task 1–3, we randomly selected 70% of the data for training, 10% of the data for validation, and 20% independent data for testing. For Task 4, due to the small number of upstaged atypia cases, we used 10-fold-cross validation for evaluation. The area under the receiver operating characteristic curve (AUC) was calculated to measure the model performance. We also calculated the specificity rates while maintaining a sensitivity rate of 100%, 99%, and 95%, respectively. These measures represent the proportion of benign or non-malignant lesions that we could potentially identify to avoid unnecessary biopsy. We used the bootstrapping methods to compute the 95% Confidence Interval (CI) [17]. DeLong’s test [18] was used to compare differences in AUC values. All statistical analyses were performed using MATLAB software, version-R2020a (The MathWorks, Natick, MA).

## Results

### Patient characteristics

Table 1 shows the key characteristics of the 847 patients. BI-RADS 4/4A/4B/4 C spans the study cohort, including 179 (21.1%) BI-RADS 4 patients, 257 (30.3%) BI-RADS 4 A patients, 217 (25.6%) BI-RADS 4B patients, and 120 (14.2%) BI-RADS 4 C patients. The mean age ± standard deviation (SD) of patients was 59 ± 12 years old for the entire cohort, with 56 ± 13 for patients with benign lesion, 56 ± 10 for patients with pure atypia lesion, 62 ± 11 for patients with DCIS breast cancer, 61 ± 12 for patients with invasive breast cancer, and 63 ± 11 for patients with atypia lesion that were upstaged to malignancy, respectively. Among all the tumor cases, 307 (67.5%) had a tumor size less than 2 cm, 71(15.6%) patients had a tumor size between 2 and 5 cm, and 11 (2.4%) patients had a tumor size larger than 5 cm. 56.2% of the patients were post-menopausal and 58.3% of the patients had a family history of breast cancer.

**Table 1:** Patient and imaging key characteristics of the study cohort

	Benign (*n* = 194)	Pure atypia (*n* = 198)	DCIS (*n* = 200)	Invasive (*n* = 200)	Upstaged atypia (*n* = 55)	Total (*n* = 847)
Age (years)$\pm$Std:	57 $\pm$13	56 $\pm$10	62 $\pm$11	61 $\pm$12	63 $\pm$11	59 $\pm$12
**Menopausal status: Number (%)**						
Premenopausal	44 (22.7)	0 (0)	30 (15)	18 (9)	24 (43.6)	116 (13.7)
Postmenopausal	41 (21.1)	65 (32.8)	165 (82.5)	175 (87.5)	31 (56.4)	477 (56.3)
Unknown/Missing	109 (56.2)	133 (67.2)	5 (2.5)	7 (3.5)	0 (0)	254 (30)
**Family history: Number (%)**						
No family history	150 (77.3)	87 (43.9)	36 (18)	45 (22.5)	7 (12.7)	325 (38.4)
With family history	38 (19.6)	110 (55.6)	154 (77)	144 (72)	47 (85.5)	493 (58.2)
Unknown/Missing	6 (3.1)	1 (0.5)	10 (5)	11 (5.5)	1 (1.8)	29 (3.4)
**Density: Number (%)**						
Fatty	8 (4.1)	1 (0.5)	7 (3.5)	1 (0.5)	0 (0)	17 (2)
Scattered fibroglandular tissue	82 (42.3)	69 (34.8)	88 (44)	88 (44)	33 (60)	360 (42.5)
Heterogeneously dense	91 (46.9)	116 (58.6)	96 (48)	93 (46.5)	22 (40)	418 (49.4)
Extremely dense	1 (0.5)	3 (1.5)	2 (1)	2 (1)	0 (0)	8 (0.9)
Unknown/Missing	20 (10.3)	10 (5.1)	7 (3.5)	17 (8.5)	0 (0)	54 (6.4)
**BI-RADS score: Number (%)**						
4	18 (9.3)	51 (25.8)	55 (27.5)	28 (14)	27 (49.1)	179 (21.1)
4A	107 (55.2)	89 (44.9)	41 (20.5)	13 (6.5)	7 (12.7)	257 (30.3)
4B	59 (30.4)	42 (21.2)	63 (31.5)	41 (20.5)	12 (21.8)	217 (25.6)
4C	6 (3.1)	12 (6.1)	26 (13)	71 (35.5)	5 (9.1)	120 (14.2)
5	2 (1)	0 (0)	0 (0)	13 (6.5)	0 (0)	15 (1.8)
Unknown/Missing	2 (1)	4 (2)	15 (7.5)	34 (17)	4 (7.3)	59 (7)
**Tumor size: Number (%)**						
< 2 cm	N/A		126 (63)	157 (78.5)	24 (43.6)	307 (67.5)
2–5 cm			34 (17)	36 (18)	1 (1.8)	71 (15.6)
5 cm			7 (3.5)	4 (2)	0 (0)	11 (2.4)
Unknown/Missing			33 (16.5)	3 (1.5)	30 (54.5)	66 (14.5)

### Model performance

**Fig. 2:**
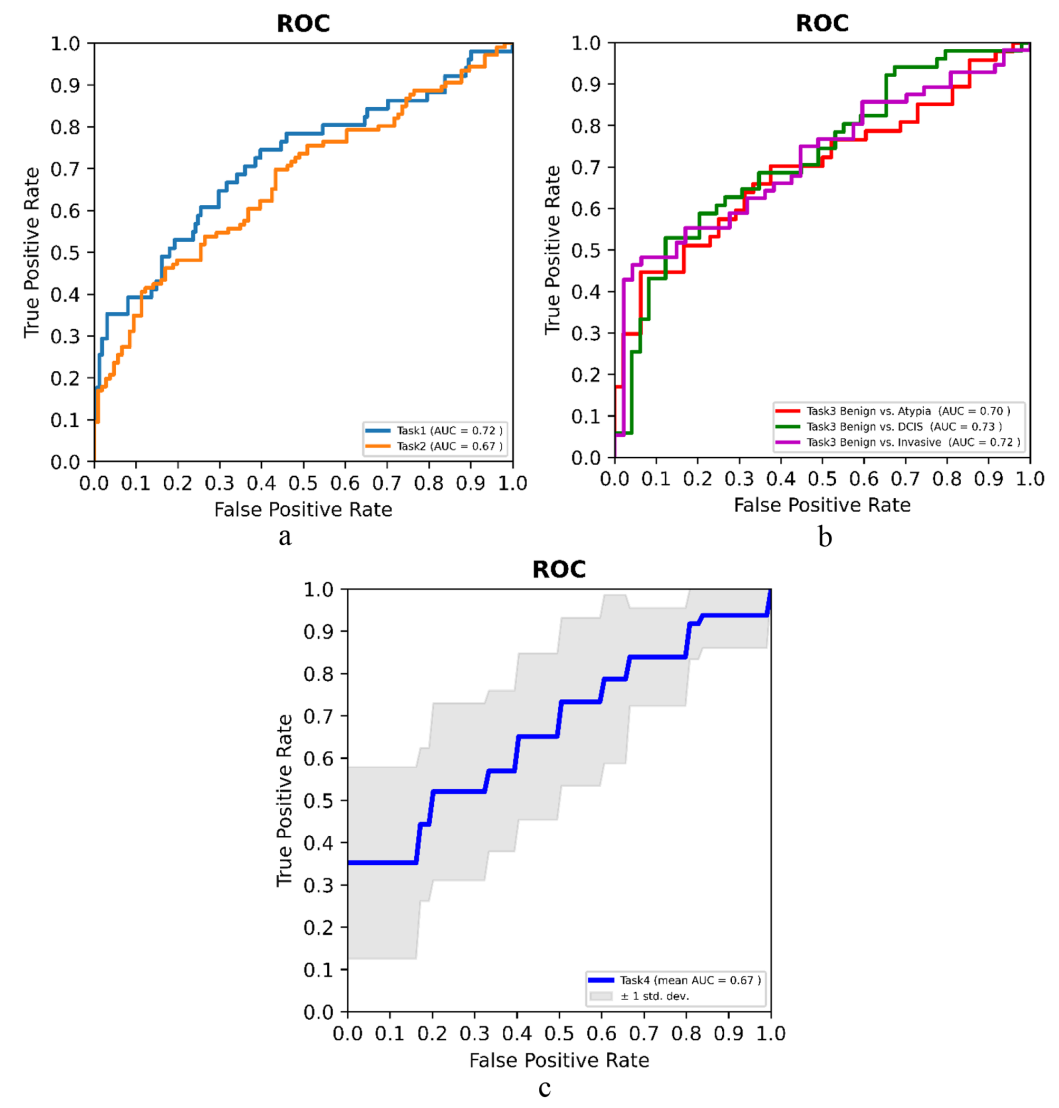
ROC curves for the biopsy outcome prediction models. Shown here are the settings with the highest AUCs. **a**) ROC curve of Task 1 and Task 2. Task 1 curve is based on CC+MLO view; Task 2 curve is based on CC+MLO view. **b**) ROC curve of Task 3. For benign vs. atypia, the curve is based on MLO view; for benign vs. DCIS, the curve is based on CC+MLO view; for benign vs. invasive, the curve is based on MLO view. **c**) ROC curve of Task 4 based on CC view

The ROC curves for the highest performance of each task are shown in Fig. 2. The results of Tasks 1 to 3 are shown in Table 2. For Task 1 (benign vs. atypia + DCIS + invasive), the AUC was 0.66 (95% CI, 0.58–0.74) using CC view images and 0.70 (95% CI, 0.60–0.78) using MLO view images. When combining CC and MLO views, the model AUC was 0.72 (95% CI, 0.62–0.80). For Task 2 (benign + pure atypia vs. DCIS + invasive + atypia with DCIS or invasive), the AUC was 0.66 (95% CI, 0.58–0.74) using images from CC view and 0.67 (95% CI, 0.60–0.74) using images from MLO view. When combining CC and MLO views, the AUC was 0.67 (95% CI, 0.60–0.74). Given a breast cancer sensitivity of 95%, the specificity for Task 1 and Task 2 was 33% and 25%, respectively. This indicates that 33% and 25% of biopsies could potentially be avoided while maintaining a 95% sensitivity for breast cancer diagnosis.

**Table 2:** AUC and specificity of Task 1–3: Task 1: benign outcome vs. all the other outcomes. Task 2: benign outcome and pure atypia outcome vs. all the other outcomes. Task 3: benign outcome vs. each of the other outcome respectively

Task		View	AUC	AUC 95% confidence interval	Specificity (%)		
					Given disease sensitivity = 100%	Given disease sensitivity = 99%	Given disease sensitivity = 95%
Task1		CC	0.66	[0.58 0.74]	5	9	25
		MLO	0.70	[0.60 0.78]	14	16	33
		CC + MLO	0.72	[0.62 0.80]	16	16	33
Task2		CC	0.66	[0.58 0.74]	7	9	19
		MLO	0.67	[0.60 0.74]	7	10	25
		CC + MLO	0.67	[0.60 0.74]	9	16	23
Task3	Benign vs. Atypia	CC	0.62	[0.50 0.73]	0	0	18
		MLO	0.70	[0.58 0.79]	17	17	30
		CC + MLO	0.68	[0.57 0.78]	17	17	30
	Benign vs. DCIS	CC	0.70	[0.61 0.79]	5	5	25
		MLO	0.71	[0.60 0.81]	7	7	30
		CC + MLO	0.73	[0.62 0.81]	6	6	25
	Benign vs. Invasive	CC	0.64	[0.60 0.80]	9	9	22
		MLO	0.72	[0.61 0.81]	5	5	46
		CC + MLO	0.72	[0.61 0.81]	5	5	43

Similarly, given a breast cancer sensitivity of 99%, the specificity for Task 1 and Task 2 was 16% and 16%, respectively. When given a sensitivity of 100%, the specificity for Task 1 and Task 2 was 16% and 9%, respectively; this indicates that without missing any breast cancer patients, our model is able to identify 16% and 9% benign patients. As can be seen, there is a slight drop in the performance of Task 2 compared to Task 1, possibly due to mixing pure atypia patients with benign patients increasing the difficulty of the classification. For Task 3, the highest AUC was 0.70 (95% CI, 0.58–0.79) on benign vs. atypia; and conditioned at the 95% sensitivity for disease, the specificity was 30%; the highest AUC was 0.73 (95% CI, 0.62–0.81) on benign vs. DCIS, and conditioned at the 95% sensitivity for disease, the specificity was 25%; the highest AUC was 0.72 (95% CI, 0.61–0.81) on benign vs. invasive; and conditioned at the 95% sensitivity for disease, the specificity was 46%.

Table 3 shows the results of Task 4. We observed a mean AUC of 0.67 ± 0.14 and the highest specificity of 35% using images from MLO view, which indicates 35% of the unnecessary excisional biopsies in atypia patients may be avoided based on the prediction of our models. Note that here the specificity remains the same at the malignancy sensitivity of 95%, 99%, or 100%, due to the small size of the samples in this task. In comparing the AUC values of the different views (i.e., CC vs. MLO view, CC vs. CC + MLO view, and MLO vs. CC + MLO view) in Tables 2 and 3, all *p*-values (ranging from 0.12 to 0.77) were not statistically significant.

**Table 3:** AUC and its standard deviation (STD), specificity of Task 4: pure atypia outcome vs. upstaged atypia outcome. 10-fold cross-validation was used for model training

Task	View	AUC (STD)		Specificity (%)	
			Given disease sensitivity = 100%	Given disease sensitivity = 99%	Given disease sensitivity = 95%
Task4	CC	0.67 (0.14)		35	
	MLO	0.58 (0.16)		18	
	CC + MLO	0.65 (0.14)		18	

## Discussion

In this study, we built deep learning models on mammogram images aiming to reduce potentially unnecessary breast biopsies. We collected a BI-RADS 4 patient cohort that consists of five categories of outcomes, namely, benign, pure atypia, DCIS, invasive, and atypia that were upstaged to malignancy. We designed four classification tasks, each with an implication for clinical considerations. We also reported the specificity of the models given a high sensitivity to measure the magnitude of potential avoidance of unnecessary biopsies. By ensuring 100% (or 99%) sensitivity of the “higher stage disease” (atypia, DCIS, and invasive breast cancer), our models can identify 5% (or 14%) of patients who may potentially avoid unnecessary core needle biopsies, and 7% (or 10%) of patients who may potentially avoid unnecessary excisional biopsies. As a preliminary work, this study proves the concept that deep learning analysis of breast mammogram imaging can provide additional information to improve the assessment of the BI-RADS 4 patients in breast cancer screening. If fully validated using larger datasets, our models may enhance clinical decision-making on breast biopsy for BI-RADS 4 patients.

Among all the four tasks, Task 1 (benign biopsy outcome vs. all the others) is the most important one to help achieve the goal of reducing unnecessary biopsy, as it is directly useful to identify potential benign lesions. The other three tasks provided additional insights for precision diagnosis. The AUC of Task 2 is slightly lower than that of Task 1, which is as expected, because the mixture of pure atypia and benign lesions as a single class in Task 2 make it more difficult for the model to learn, especially when the sample size is not large. Task 3 (benign vs. each malignancy outcome) provides a closer look at distinguishing the several subcategories, where the AUCs show that it is harder for machine learning to distinguish benign vs. atypia, compared to distinguishing benign vs. DCIS and benign vs. invasive cancer. For Task 4, the AUC is relatively low, which may reflect the difficulty of learning further nuances of imaging features of initial atypia lesions, and/or the limitations of the smaller sample size for this task.

Looking at different views of the mammogram, we observe that both CC and MLO views have predictability for all the tasks, indicating both views contain information related to the biopsy outcome. In Task 1, Task 2, and Task 3, the MLO view had slightly higher AUCs than the CC view, but the differences were not significant. For Task 4, it is the opposite that the CC view may be more related to predicting excisional biopsy outcome than the MLO view. In general, combining CC and MLO views increased AUC values but did not reach statistical significance, which may also have to do with the sample sizes. The effects of CC and MLO views merit further investigation in future work.

Some previous studies pertaining to BI-RADS 4 lesions have been reported, including using breast MRI [19], plasma microRNA [20], proteomic biomarkers [21, 22], etc. Henderson and colleagues showed by integrating breast serum proteomic markers into the clinical analysis process, 45% of unnecessary biopsies in BI-RADS 4 lesions may be spared. It should be noted that 204/540 benign subjects in that study were not biopsy-proven [23]. Tiancheng He and colleagues [24] built a biopsy decision support system for BI-RADS 4 lesions using deep learning, where both atypia and lobular carcinoma in situ (LCIS) were classified as “benign”. In most clinical settings, patients with atypia and LCIS may still require biopsy or may be candidates for possible chemoprevention using anti-estrogen therapy [25]. It is thus critical to distinguish pure atypia from atypia that are upstaged to malignancy. In our study, the deep learning models may identify 35% of the pure atypia that may potentially spare excisional biopsies conditioned on 100% sensitivity.

In addition to digital mammograms, it will be also important to explore how other breast imaging modalities and/or clinical variables may influence the accurate assessment of the BI-RADS 4 patients. For example, additional ultrasound screenings have demonstrated improved detection rates for breast cancer [26]. Digital breast tomosynthesis depicts multiplicity of some masses that may otherwise have been unnoticed in other image modalities [27]. Some of these imaging modalities are often performed before biopsy and thereby they may provide additional information to improve the performance of the proposed deep learning models. In future studies, we plan to build advanced machine learning models using multi-modal imaging data and clinical variables.

Our study has limitations. First, the dataset was collected retrospectively from a single institution and the sample size is relatively small. This preliminary study is to prove the concept, and it is warranted to further evaluate our models using external and larger patient cohorts to increase the generalizability across different practice types and screening populations of different regions. Our study cohort is representative of the local screening population of our region, but we notice that the mean age of our cohort is ∼ 60 years old, which may suggest lower breast density and possibly higher sensitivity of mammography, when compared to the age group of 40–50 years old women in the screening population. In addition, we employed the simple VGG16 network in our deep learning model backbone for this preliminary study. More sophisticated deep learning modeling techniques may further improve the model performance. Finally, following a similar approach to examine the effects of digital breast tomosynthesis will be worthy of further investigation.

In summary, this study shows that deep learning models on mammogram images can classify breast biopsy outcomes for BI-RADS 4 patients. While this is a preliminary study that needs further evaluation, it shows the deep learning approach has the promise to improve decision-making for breast biopsies to potentially reduce unnecessary biopsies and the attendant costs and stress for the BI-RADS 4 patients.

## Data Availability

The medical imaging data used in this study are not publicly available due to patient privacy considerations. Interested users may request access to the data for research purposes, through contacting the corresponding author. Institutional approvals of data sharing will be required along with signed data use agreements and/or material transfer agreements.
